# Information Needs and Data Harmonization—Two Sides of the Same Coin?

**DOI:** 10.5334/dsj-2025-030

**Published:** 2025-10-31

**Authors:** A. J. MILLION, JENNY BOSSALLER, SANJA GIDAKOVIC

**Affiliations:** University of Michigan, Inter-university Consortium for Political and Social Research, Ann Arbor, MI, USA; University of Missouri, School of Information Science & Learning Technologies, Columbia, MO, USA; University of Missouri, School of Information Science & Learning Technologies, Columbia, MO, USA

**Keywords:** data harmonization, firearm-injury prevention, information needs

## Abstract

This article reports on a quality improvement project aimed at supporting the Community Firearm Violence Prevention (CFVP) network at the University of Michigan in the U.S. We examine the information and data needs of researchers in public health, medicine, and criminal justice. Semi-structured interviews with CFVP affiliates and researchers confirm the existence of significant limitations in existing datasets and resources to conduct firearm-violence prevention research. Study participants discussed (1) the infrastructure and research support that would improve their productivity; (2) benefits of using and combining datasets, (3) challenges when accessing data, and (4) data collection and harmonization strategies that increase research impact. We present findings highlighting data-related challenges in this space that concern researchers, data scientists, and policymakers. We conclude that researchers’ information needs and data harmonization initiatives are interrelated phenomena. Our observation carries implications for large-scale data harmonization efforts in the social and behavioral sciences.

## INTRODUCTION

In 1996, the U.S. Congress passed an appropriations bill that included the *Dickey Amendment*, which prohibited the use of federal funds to promote or advocate for gun control. The amendment targeted political advocacy, but the result was a *de facto* ban on funding all research related to firearm injuries and fatalities. A presidential memorandum later clarified that the *Dickey Amendment* does not ban research about firearm injury prevention. Congress agreed, and between 2020 and 2022, the CDC and the National Institutes of Health (NIH) awarded more than $150 million for firearm injury-prevention research researchers (Lin et al., 2024). However, the decades-long funding moratorium also produced a ‘disordered and highly segmented’ data ecosystem that was useful for only ‘narrow studies to inform national policy and for use in local operational decision-making’ (Roman, 2020b, p. 2).

In 2019, researchers at the University of Michigan created the Firearm Safety Among Children and Teens (FACTS) Consortium. FACTS provided a foundation for the Community Firearm Violence Prevention Network (CFVP) that collects, evaluates, and archives data produced by research teams that study interventions and community factors contributing to firearm-related injury and death.

This paper reports on a quality improvement project aimed at informing and extending the CFVP by examining the information and data needs of public health, medical, and criminal justice researchers. Semi-structured interviews with CFVP affiliates and researchers confirmed the existence of significant limitations to existing datasets and supplemental resources. Study participants discussed (1) the infrastructure and research support that would improve their productivity; (2) benefits of using and combining datasets, (3) challenges in accessing data, and (4) data collection and harmonization strategies that increase their research impact. We present findings that highlight data-related challenges in this space that are widely applicable to researchers, data scientists, and policymakers in the social and behavioral sciences.

## LITERATURE REVIEW

Here, we provide background information about the CFVP network. We also review the literature on harmonization as a strategy for maximizing the utility of data, survey current firearm injury prevention (FIP) research, and describe the data infrastructure used by researchers. By infrastructure, we specifically mean the people, systems, networks, and institutions that allow us to ‘generate […] knowledge about the human and natural worlds’ (Edwards, 2013).

### COMMUNITY FIREARM VIOLENCE PREVENTION NETWORK

The CFVP Network (https://endfirearmviolence.org/) supports research that develops and tests behavioral and medical firearm-related interventions. The network consists of four groups: (1) data and methods; (2) community engagement; (3) policy, implementation, and economics; and (4) communication and dissemination. As of November 2024, the network supports six projects located across the country with a range of intervention strategies (e.g., providing legal guidance to victims). The data and methods group’s tasks include aligning study measures with one another and creating research data management practices that ‘enable robust cross-project analyses that accelerate the science of preventing firearm injuries’ (Sokol et al., 2024, p. 1122). Since research in this area has only recently returned, CFVP began with the goal of harmonizing measures and study results across sponsored projects. Sokol et al. (2024) delve into the benefits of CFVP’s approach and observe that even rigorous studies may not be generalizable, fail to measure key phenomena, or lack adequate data to assess the effects of interventions. Meta-analyses, too, are sometimes hindered by incomparable measures, varied timelines, and limited data (p. 1123). To circumvent these issues, the CFVP requires all six NIH-funded projects to collect data using a harmonized codebook at two assessment points.

### DATA HARMONIZATION

Data harmonization refers to a research process that allows researchers to compare data across varied sources and formats. In their primer on data harmonization efforts for COVID-19, Cheng et al. (2024) argue that harmonization has three dimensions: *syntax*, *structure*, and *semantics*. Syntax refers to data formats (e.g., CSV and HTML files) that require processing before it is possible to harmonize datasets. Structure, or conceptual schema and the organization of data points, describe how ‘variables relate to each other within a dataset’ (p. 2). Semantics describe the ‘intended meaning of words’ and symbols that comprise a dataset.

A broader definition of *data* includes

Geospatial coordinates, numerical values, and measurements… literature corpora, images, or physical samples [that may…] provide evidence of phenomena or to serve as a subject of analysis [and which] can be organized in different ways (i.e., in spreadsheets, as networks or graphs, or as collections of related artifacts) (Million et al., 2025, p. 2).

Homogeneity across datasets means that harmonization may succeed in one context but fail in another. Cheng et al. (2024) treat syntax and format as synonymous, but information scientists distinguish between the two.

Despite challenges in harmonizing data on a grand scale, we know that harmonization is effective. The Helping to End Addiction Long-term (HEAL) Prevention Cooperative (HPC) has utilized data harmonization to examine a range of evidence-based interventions aimed at preventing opioid misuse (Ridenour et al., 2023). Each of the 10 HPC projects has distinct characteristics (e.g., intervention strategies, theoretical models, settings, and populations) related to opioid misuse. The U.S. Institute of Museum and Library Services (IMLS) sponsored a harmonization effort in 2022 to evaluate public library programming outcomes (Adkins et al., 2023). CFVP’s harmonization effort bridges and combines research, and the number of harmonization-based research applications is growing due to changing scientific norms and requirements for data sharing (Nelson, 2022), as well as the ‘deluge’ of data (Hey and Trefethen, 2003) available to researchers.

### FIREARM INJURY-PREVENTION RESEARCH AND INFORMATION NEEDS OF RESEARCHERS

As suggested earlier, the opportunity for research teams to utilize federal funding for FIP research presents new avenues for identifying patterns of violence and testing interventions aimed at reducing violence and promoting safety. The passage of the *Dickey Amendment* and the subsequent moratorium on funding resulted in a decline in peer-reviewed FIP research articles, starting in 1996 (Galea et al., 2018). Recent work focusing on FIP has set agendas for an entirely new generation of research. Cooper et al.’s (2022) survey found that FIP researchers rate suicide (29.6%), gun violence (20.1%), and violence prevention (18.3%) as the most important topics to study. During the COVID-19 pandemic, FIP research emphasized topics such as domestic violence, firearm storage, stay-at-home orders, and suicide (e.g., Anestis et al., 2021; Duncan et al., 2020; Lyons et al., 2021). Many researchers equate FIP and automobile safety research, which contributed to a 90% reduction in deaths per vehicle mile driven in the U.S. Automobile safety was achieved through behavioral, engineering, policy, and cultural changes (Roche et al., 2023, p. 583) at all levels of society, and FIP researchers replicating the strategy employed by auto safety researchers hope to produce similar outcomes.

One purpose of our project was to ensure that the CFVP properly supports and meets the information needs of FIP researchers. By *information need*, we mean the ‘information objects’ (e.g., datasets and textual documents) required to conduct rational, empirically valid research (Buckland, 1991; Wilson, 2006). Case and Given (2016) note that online information has alleviated concerns about information access for many scientists, but social networking, gatekeeping, and interpersonal knowledge sharing—the ‘invisible college’—remain crucial. They found that social scientists frequently utilize administrative data. Healthcare providers need to be aware of research findings and their patients’ conditions (p. 253), so they consult textbooks, drug guides, and other resources in applied settings. Scholars who work ‘between disciplines,’ like our interview participants, whom we discuss later, operate in a ‘scattered’ information environment (p. 247).

### DATA INFRASTRUCTURE

Despite an increase in funding for FIP research, anecdotal reports from CFVP stakeholders suggest that data remains scattered, unavailable, or are only accessible in sites with limited access. Public health researchers aim to identify the underlying causes of population health issues to inform targeted behavioral and medical interventions. These researchers rely on accessible, accurate data.

FIP research has long been hindered by structural barriers, including a lack of funding and missing or low-quality data (Jamieson, 2013). FIP researchers also need access to data describing communities, as well as information about the social, psychological, and behavioral levels of phenomena. Magee (2023) finds that firearm injury and fatality data are rarely accessible at the neighborhood level. A series of reports by the National Opinion Research Center (NORC) describe the fragmented state of America’s firearm data infrastructure (Roman, 2020a, 2020b, 2020c; Roman and Cook, 2021). These reports find that data are scattered across sites that could function during the funding moratorium, including the Gun Violence Archive, the Centers for Disease Control and Prevention (CDC), the RAND Corporation, Boston University, the American College of Surgeons, and state administrative systems. Data about injuries, available from hospital trauma units, document psychological and social factors that drive firearm violence and injury (Richardson, St Vil and Cooper, 2016). However, it is not hospital workers’ responsibility to clean and disseminate these data.

FIP researchers also need data generated using consistent definitions and reporting procedures, but neither is always supported by existing systems. Gobaud et al. (2023) assessed Gun Violence Archive (GVA) data from four US cities and found it was useful for studying urban areas. However, systematic biases and missing data, including data on nonfatal shootings, limit its research utility. To identify areas of overlap between the GVA and official records, Magee (2023) studied ‘the Nonfatal Shooting Review database, which is generated using information from both police incident reports and internal documents’ (p. 303) in Indianapolis, Indiana. The CDC’s National Violent Death Reporting System includes fatality data missing from Indianapolis, but it provides limited geographic information (Barber, Cook and Parker, 2022). Durkin et al. (2020) note that states collect consistent data from ‘crime reports and public health systems’ (p. 33). However, because each state has its own needs and priorities, the quality of data varies.

## METHODOLOGY

To support FIP researchers, the CFVP partnered with ICPSR to create an online collection of data. ICPSR is a well-established data archive that houses over 10,500 social science studies, comprising more than 255,000 data files across 17,750 data collections contributed by researchers, research centers, and governmental agencies. ICPSR maintains a web-based catalog that supports ‘faceted’ data searches using metadata standards and controlled vocabularies. However, given the state of the art regarding FIP research, the information needs of researchers are not well understood. Thus, we asked three research three questions:

**RQ1**: What are the information needs of CFVP stakeholders?

**RQ2**: What data-related problems were expressed by the people we interviewed?

**RQ3**: What points of agreement exist about firearm violence and injury-prevention data among the people we interviewed?

### NETWORK STAKEHOLDERS

To conduct this study, we interviewed CFVP stakeholders in three groups:

- Healthcare and public health researchers (e.g., sociologists and statisticians) affiliated with the CFVP,
- CFVP staff and administrators, and
- FIP criminologists who are not affiliated with the coordinating center, but whose research needs and experiences we sought to compare to those of public health researchers.

These groups possessed diverse perspectives about the needs of, and issues faced by, FIP researchers. Public health and medical workers are the primary contributors to CFVP intervention evaluation studies and work closely with the center’s staff. CFVP staff were positioned to discuss the relationships among research studies and the broader FIP research landscape. Criminologists mostly relied on crime data and did not face the same problems as public health researchers during the funding moratorium.

We interviewed 15 people: five *researchers working on CFVP-supported projects*, four *CFVP faculty and staff members*, and six *criminologists* identified through a review of FIP research projects in Google Scholar.^1^ The researchers were multidisciplinary scholars who used diverse data types and research methodologies. Six participants worked in *public health*, four in *crime and criminal justice*, two in *biostatistics*, and two in *public administration*. *Biopsychology*, *community psychology*, *economics*, *education*, *nursing*, *organizational psychology*, *pediatrics*, *psychology*, *quantitative psychology*, *social work*, *sociology*, and *trauma surgery* were each represented by one researcher. Seven used *mixed methods*, and five were *quantitative researchers*. Two reported that they used *computational methods*, and two were *experimental researchers*. Nine stated that they relied on *secondary data* for their research, while six used *original data* they collected.

We did not employ a formal sampling approach when selecting interview participants, because our project’s goal was not to produce generalizable scientific knowledge—it was a user study. We elaborate on this point in the section below. However, we did recruit what might be called a *maximum variation sample* of individuals ‘to document unique or diverse variations’ among FIP researchers (Palinkas et al., 2015; Patton, 2002). Central to our effort was the goal of developing a resource including archived and indexed FIP datasets. To develop this resource, we needed to understand FIP researchers’ information needs.

### SEMI-STRUCTURED INTERVIEWS

Because this was a quality improvement (QI) project, our goal was not to collect generalizable information but to support CFVP stakeholders. At least two interviewers conducted each interview, providing participants with opportunities to elaborate on their responses and address unanticipated topics that arose during interviews. Each interview began with an explanation of the study’s purpose and a clarification that the project did not constitute ‘human subjects research.’ For good measure, and to align with good research practice, we asked for participants’ consent to participate in, record, and then transcribe our interviews. Next, we asked each researcher about their discipline, the type of research they conduct related to FIP, the research methods they have used in this work, the data that would enhance their research, and data-related problems they have faced in the past (e.g., availability, cost, and documentation). Next, we asked participants to envision an ideal data resource for their work and the support they needed from CFVP to succeed (e.g., help accessing restricted or sensitive data). Our participants did not represent all FIP researchers, but given the small size of our research community, our findings were sufficient. See Appendix A for the interview questions that we asked.

We used an *investigator triangulation strategy*, adapted from Lincoln and Guba (1985; as cited in Korstjens and Moser, 2017), to increase the trustworthiness of our analysis. This required all three authors to participate in the coding, analysis, and interpretation of the interviews. Two authors conducted the initial thematic analysis by reading through each interview multiple times and identifying common themes to address our research questions. When reading interview transcripts, we created memos that noted participants’ experiences and identified relevant quotations. Next, we reviewed the interviews as a group, assigning thematic labels to highlighted passages. We followed this iterative process until no new information emerged. Finally, the third author reviewed the codes, conducted spot checks, and synthesized themes in an Excel spreadsheet to confirm our analysis. The result was an organized set of textual passages from domain experts that we used to answer our research questions.

## FINDINGS

There were eight themes in our interviews. In this section we discuss these themes. See Table 1 for these themes and interview passages that we provide as examples of what the themes describe.

### SOME FIP RESEARCH PROBLEMS ARE NOT UNIQUE

Many of the problems that our interview participants described are common. We found that (1) controlling for confounding variables is challenging, much like in other research domains. Our 15 interview participants also said (2) valuable data, including datasets with key measures (e.g., an individual’s history of mental health issues) are not always reported in official data; (3) laws and regulations, such as the Health Insurance Portability and Accountability Act (HIPPA) impedes data collection for research (albeit for good reasons); and (4) data collection is time consuming. For example, two criminologists discussed using U.S. Federal Bureau of Investigation (FBI) data. Closed cases are available through Freedom of Information Act (FOIA) requests; however, case records can be thousands of pages long. Obtaining the data can be time-consuming, and officials can only provide data as resources permit. Complicating matters, FOIA requests require researchers pay government agencies for coarsened datasets, so many researchers scale back their work, use low-quality data, or abandon projects altogether.

### MANY TYPES AND SOURCES OF DATA EXIST

Another finding relates to differences in data types and their sources. We asked researchers where they found data and whether they collected it themselves. Some mentioned the complicated nature of FIP research, explaining that taking a national picture of firearm violence requires data from many organizations and perspectives, including from different disciplines. For instance, hospitals collect the medical history of victims. Social scientists (e.g., sociologists and criminologists) need data about social risk factors, such as individual socioeconomic status, education, sexual orientation, race, and age. Social workers have a broader perspective on victims’ trauma and adversity, so their insight is valuable, but it is typically stored in unstandardized case notes. Police and FBI data include extensive information about offenders, including crime details, perpetrator motivations, and victim information. The CVFP network exemplifies how interdisciplinary collaboration can bridge fragmented data sources—medical, social, and criminal justice—to generate a more comprehensive understanding of firearm violence. However, it is worth noting the expansive scope of researchers’ information needs.

Some researchers whom we interviewed discussed their need for specific information (e.g., ‘was an injury self-inflicted or related to domestic violence?’). Authorities do not always collect these data, so they are not uniformly available. There is also missing information about nonfatal injuries because some victims do not seek medical care, or their wounds might not be recorded as gun related.

Differences among geopolitical jurisdictions indicate a need for baseline, shared measurements to conduct national research. One participant said that they have been ‘trying for years’ to obtain access to gun violence data in their state, but Colorado, ‘very much a gun rights state,’ employs a data scientist. ‘They have all the linked data—everything,’ including SNAP eligibility, type of injury, prior criminal histories, and other factors. However, all state governments need the funding, expertise, and motivation to make that happen. Another participant pointed out that ‘there is a strong difference in the policies that might have an uptake in red states versus blue states.’ Clarifying this statement, they said:

We don’t have a gun epidemic in the US, we have 51-gun epidemics in the US. And, they’re all different. Utah [has] more suicide, Mississippi [has] more homicide… [yet] when we have a discussion about gun injury and violence, it’s using very national descriptions and flavors.

These examples highlight the need for standardized yet adaptable data systems that account for the diverse policy environments and patterns of firearm violence across American political jurisdictions.

Participants discussed 43 sources of data. Table 2 lists these sources, categorized by subject (e.g., crime and criminal justice), which reflect the fragmented nature of the U.S. national firearm data infrastructure. Several researchers collected or used qualitative data, and quantitative researchers agreed that qualitative data is essential for understanding the root causes of violence, such as individuals’ motivations and treatment outcomes for interventions. However, these data are often not suitable to draw generalizable claims, which makes them less suitable for secondary use.

### CREATIVELY COMBINING DATA CAN CAUSE PROBLEMS

Combining datasets can reveal new findings, and three of our interviewees described creatively ‘Frankensteining’ data. However, they also recognized the problems that occur when using data from outside their academic disciplines. It is not enough for researchers to use data to generate factual knowledge; researchers must also understand the data, including its limitations and idiosyncrasies, as well as the methods used for data collection, imputation strategies, and the underlying phenomena being measured. Without understanding data, individuals or larger teams of researchers can easily misrepresent statistical findings or draw erroneous conclusions.

One example of a researcher expressing concerns about the combination of data related to a multi-measure instrument, the Conflict Tactics Scale (CTS), which is provided by Straus et al. (1996). This instrument provides a way to describe violence involving intimate partners, and one interview participant adopted and expanded the CTS for their work. However, they also said

I feel like the lack of [established] measures is the biggest problem for firearm research… we’re taking a lot of the measures… [but a] lot of it, I call it Frankensteined. It’s like taking pieces of things and sewing them together in a different way and adopting them.

A lack of established multi-variable measures relating to firearm injuries prompted this researcher to acknowledge the field had much more to learn. Unfortunately, a lack of knowledge about ideal measures also made it difficult for researchers to collect the data they might need in the future.

Existing databases change over time. Researchers sometimes add new questions to study questionnaires in healthcare settings, and lawmakers may mandate research on pressing issues. Regardless of how researchers measure data, one of our participants made an important point: incorporating new questions in large-scale data collection instruments ‘in a way that doesn’t completely fracture the structure that was already created’ can be a challenge unto itself. One criminologist discussed the Bureau ofJustice Statistics’ National Crime Victimization Survey and the National Incident-based Reporting System, noting that both have changed over time. The NCVS includes multiple questions pertaining to cargo theft and cybercrime because the U.S. Congress wanted to gather information about these crimes. However, by adding these questions, the government also confused people used to prior versions of the survey data.

### POLITICAL CONSTRAINTS ARE OFTEN MANAGEABLE

Several of our interviewees indicated that state and national politics can pose barriers to conducting research about the causes and correlates of firearm violence, but typically, these barriers were manageable. Elected officials interpret the purpose of the 2nd Amendment of the U.S. Constitution differently, and some researchers said that *any* research examining firearm violence has the potential to become political. However, others said their work was supported by everyone because they only studied violence prevention. A more significant political issue related to data from police departments and local governments. Five study participants emphasized the importance of establishing relationships with government agencies. Building trust to obtain data successfully was a political process because it required demonstrating an ongoing commitment to protect departments against unfair criticism, ensuring that data was used for agreed-upon purposes to promote the common good.

### NOT ALL RESEARCHERS WANT TO SHARE THEIR DATA

While the Open Science movement has widespread support in academia and government, many researchers we interviewed expressed concerns about sharing the data they collect. Successful data reuse depends on both the quality of the data collected and the quality of accompanying documentation. While there was broad recognition that some data cannot be freely shared because of confidentiality and privacy issues, some of our participants discussed problems with sharing data because they wanted more time to analyze it. Interviewees were sometimes concerned about being ‘scooped’ by other researchers or having their findings misinterpreted or manipulated. Other participants mentioned being unsure of the best places to store sensitive data. As they pointed out, some (but not all) grants specify archiving study data in a repository. They felt it was essential to manage how data would be accessed, by whom, and to protect it from loss.

### DATA HARMONIZATION IS CONTENTIOUS

Another challenge noted by some participants was the need for data harmonization across CFVP study sites. Fields such as psychology have freely available, standardized measurement scales, but those are generally lacking in FIP research. A CFVP staff member underscored the importance of standard measures and the need for ‘a compendium of measurement tools in a website […] that people could pull quickly.’ They said it is easy to do this with many mental health measures, but firearms data is more complicated, because the field is only now emerging.

Validating measures takes time and can be complicated by socioeconomic and other confounding factors. Furthermore, some research constructs are difficult to define in binary terms. For instance, one person raised concerns about survey questions regarding firearm storage. A gun owner might keep theirs in a locked case at home, but if it is carried, its status might change throughout the day:

What does safe storage mean? Does that mean 100% storage, it never comes out of the safe, or it only comes out of the safe when it’s unloaded […] and then it’s in the trunk of my car? So, thinking through some of the measurement-related issues with firearms, I think is really important. How do you combine those factors? And then there’s handguns and long guns… that adds another component of complexity.

The codebook for the CFVP network was created during a preliminary phase of research at individual study sites, well before data collection commenced. Some sites aspire to conduct research based on new topics that emerge during their research, that is, after the codebook was created. The result was problems with requirements issued by the coordinating center when balancing standardization and project autonomy.

Similarly, a data manager at the coordinating center described the data they are collecting, which includes demographics, social determinants of health, mental health, and substance use. Their data falls under multiple domains, including firearm violence, physical health, and protective factors. Within these domains are multiple constructs. Anxiety, depression, loneliness, and PTSD are measured using standardized tests. Standardized measures helped CFVP staff harmonize data, but not all researchers wanted to use those measures.

CFVP staff emphasized that project Principal Investigators (PIs) also need to understand how their data might be used in the future and how they want it curated for reuse. The PIs wanted clean data that was compatible with statistical analysis software. Collecting data on the same platform (e.g., in a uniform database) would streamline the data collection process, ensuring some degree of fidelity to a data codebook. However, researchers wanted to be able to shape their projects as desired. One individual said, ‘People underestimate the amount of work it takes to make a dataset publicly available and useful to other researchers’ because of the hidden, invisible labor associated with processing it.

### THE NECESSITY OF GRANTS

Another prominent theme in our interviews, mentioned by eight participants, was the need for grant funding to support research, particularly in relation to the tenure and promotion process. Our participants were at various stages of their careers, and securing grant writing and external funding was critical for untenured researchers. During the period when funding for firearms violence research was scarce, researchers turned to other subjects (e.g., substance abuse and youth violence prevention). One participant was advised by a mentor to avoid FIP research because focusing on it before 2019 had been a ‘career killer.’ A younger researcher, beginning their career just as federal funding was restored, interpreted congressional guidance for firearm-injury research as an opportunity and chose to study the topic. Our study participants agreed that federal grants were central to FIP research because they are the only widespread funding source for researchers.

### COMMONALITIES: NEEDS SHARED AMONG RESEARCH ENVIRONMENTS

Our final finding was a set of agreed-upon points that interviewees believed were necessary to support firearm injury-prevention research. Researchers indicated that they needed rich, clean, and comprehensive data with correct and uniform variables. Data collected and shared by hospitals, police, social workers, and other parties must be ‘open’ and provided by, or at least indexed within, easily accessible look-up resources. Databases, datasets, and other information resources must include social risk factors (e.g., prior convictions related to gun violence and sociocultural factors). Participants noted the need for different variables, depending on their academic discipline and research requirements. Some participants proposed a comprehensive state data hub or dashboard of public data that was well-maintained and monitored by a public agency (e.g., the CDC), which could be used across various jurisdictions to demonstrate relationships between variables and outcomes. That type of dashboard would have established baselines, allowing longitudinal studies to demonstrate the effects of medical and behavioral interventions.

Over half of our participants talked about the need to harmonize and standardize measures across different CFVP projects. Social risk factors (e.g., food insecurity, housing, and underemployment) were important constructs for researchers to measure. One interviewee said

the reason we really want to have some unified measures that are standardized or harmonized across the network projects is because there are so many questions that we as a field need to answer. And many of those questions we are not [statistically] powered to answer within the context of the single study. But if we have the same question asked among three times or six studies, maybe we would have the power to ask and answer some of the questions we have.

This quote highlights the importance of using standardized measures across CFVP projects to facilitate cross-study comparisons.

Geographic granularity was also a necessary data attribute for many of our interviewees. While some individuals said that they simply wanted to have data available at the national level (e.g., the number of firearms in each county), their ideal research environment and ‘stack’ of supplemental resources included information that described states, localities, and even ZIP codes. Regarding geography, interview participants discussed the need for uniform reporting across political jurisdictions. A lack of uniformity across the country made it difficult to draw comparisons or reach conclusions about the effectiveness of interventions. Combining temporal, geographic, and socioeconomic data and isolating relevant variables could also enable individuals to develop targeted interventions in high-risk areas.

Our findings about geographic data also extended to questions about the timeliness and currency of data. Some research projects that our participants spoke about (e.g., behavioral interventions) were only possible when researchers recently collected data. Several interviewees expressed frustration that publicly available data was released 2 years after a given period ended. While older data can be used to answer theoretical questions, write background sections for grants and papers, and take a long view when describing social and behavioral phenomena, timely datasets are needed to address contemporary issues.

## DISCUSSION

Throughout this project, we sought to answer three questions that would inform CFVP operations and help us to design data resources that facilitate FIP research. That said, a more significant finding was that researchers’ information needs and data harmonization are two sides of the same coin. We also determined that FIP researchers can make discoveries by methodically organizing and collecting new data. There were impediments to their work, to be sure, but during our interviews, we were surprised by how simple impediments to research appeared—we began this project assuming FIP was a completely unique research space, and we concluded the opposite. We now believe that most FIP data issues stem from long-term neglect by researchers and federal underfunding.

### WHAT ARE THE INFORMATION NEEDS OF CFVP STAKEHOLDERS?

Contrary to our expectations, although some CFVP stakeholder information needs were unique to firearm violence prevention, most were not. Because the information needs of CFVP stakeholders were transferable to most other domains, we interpret our results to mean the answer to RQ1 is that researcher information needs are comparable to those of others, particularly in the social sciences.

The data needed by firearms violence researchers is complex, varied, and interdisciplinary. As framed by Case and Given (2016), FIP research is between disciplines. Furthermore, firearm violence, like many other public health issues, is not contained within political borders, yet local jurisdictions have very different policies, motivations, and abilities to collect, process, and store both hospital and police administrative data. To perform their work effectively, FIP researchers require data that pertains to social services, law enforcement, and health factors. It should also be curated (or at least indexed) in public repositories.

Although the data-related issues we identified were not unique, we did identify unique contextual challenges that are worth mentioning. For example, our findings corroborate Roche et al. (2023), who present five issues in studying gun violence:

… inadequate funding; a lack of comprehensive data availability and access; limited evidence-based programming; limited diverse and scientifically trained researchers and practitioners; and the stigma, polarization, and politicization of this field of study (p. 583).

Relatedly, some of the data problems our interviewees discussed included a lack of research funding to support scholarship and political constraints. Both contributed to a dearth of data and a lack of influence on data-sharing practices. A historic and lingering lack of political will to collect data (by hospitals and police) and fear of political retribution or withdrawal of funding made data both expensive and difficult for researchers to obtain. They also discussed the lack of coordination between states, which suggests that a consortium or network led by data managers, encompassing multiple states or regions, would be a significant improvement. However, to promote cross-organizational efforts, hospitals, coroners, urgent care centers, and law enforcement agencies all require training, political will, and funding to collect and report data for this cause.

### WHAT DATA-RELATED PROBLEMS WERE EXPRESSED BY THE PEOPLE WE INTERVIEWED?

Although most firearm-injury prevention research problems are not unique, our answer to RQ2 is somewhat more nuanced. As shown in Table 2, a variety of types and sources of relevant data are already available to researchers. Fragmentation in these resources presents a significant challenge that is unique to FIP research. The state and composition of these resources (given the political reality associated with decades of limited funding and research prioritization) limit their utility, and we believe this stems from the necessity of grant-funded research, even though, beyond this point, the problems expressed by CFVP stakeholders were still like those in other fields.

Perhaps the most concise description of the data-related problems we identified can be addressed by the FAIR Principles. The FAIR (*Findable*, *Accessible*, *Interoperable*, and *Reusable*) Principles offer a set of guidelines and have been widely adopted, including by biomedical researchers (Wilkinson et al., 2016). Biomedical research is expensive and often funded by federal agencies and organizations that increasingly require researchers to develop data management plans. Some researchers have criticized the FAIR Principles because certain data should not be shared, particularly when governance, confidentiality, and ownership issues are involved. Our interviewees believed that making their data completely ‘open’ would violate the trust they had built with individuals who provided it. However, this view reflects a misconception of the FAIR Principles, which do not suggest that all data be public. Rather, what matters is that datasets are discoverable and described with rich metadata, including clear indications of local access restrictions (Landi et al., 2020).

There are many different reasons why individual researchers and organizations do not share data according to the FAIR Principles. For instance, (1) criminologists sometimes pay large sums to obtain data from police departments or the FBI using FOIA requests, so they do not want to share it with others for free. Furthermore, (2) health researchers cannot viably remove identifiable data from patient records, and organizations have a legal responsibility to protect subject confidentiality and privacy. Third, (3) many researchers do not want to be ‘scooped.’ They might prevent this by imposing an embargo on releasing data. Collectively addressing many of these issues cannot be done by individuals.

### WHERE WERE THE POINTS OF AGREEMENT ABOUT FIREARM VIOLENCE AND INJURY-PREVENTION DATA AMONG THE PEOPLE WE INTERVIEWED?

The points of agreement we identified among interview participants (RQ3) primarily stemmed from efforts to harmonize data collected with support from the CFVP, and these may provide lessons for attempts to redevelop and streamline America’s firearm data infrastructure. The CVFP project aims to harmonize data from six studies and has identified considerable overlaps between projects by establishing a shared codebook and a set of measures for researchers to use. However, these teams still depend on supplemental resources to succeed. In our work, we found that the shared resources FIP researchers need are complex.

Beyond the practical problem of finding data, some participants described issues with others’ interpretation of data from outside their field; they had found studies that inaccurately reported data, having been creatively (but improperly) combined from multiple sources due to researchers’ misunderstandings. These authors may also not have read the ‘fine print’ about how secondary data was processed. A shared set of resources, including a harmonized dataset in an archive, such as ICPSR, resolves this problem. However, researchers still needed access to data from sources that will never be collected, curated, and stored by the same entity or organization. Data harmonization and leveraging information from multiple sources are not mutually exclusive activities but doing both at the same time is sometimes impractical for cost-related reasons.

Project-specific data harmonization efforts, such as the Helping to End Addiction Long-term (HEAL) Prevention Cooperative (HPC) and the data module for the Public Library Association’s Project Outcome initiative (Adkins et al., 2023), provide roadmaps to combine datasets when FIP researchers choose to harmonize datasets. The advantage of including multiple data types and resources is obvious: researchers can cross-reference and identify phenomena more easily and accurately. However, there are obstacles that all researchers face. Data are not uniformly collected across jurisdictions, and different methods of imputation and statistical maneuvering affect how data can and should be used.

Data harmonization was the primary strategy used by the CFVP network to help researchers work together, and it featured prominently in our interviews. However, even the small group of researchers affiliated with CVFP described challenges (i.e., establishing points of agreement regarding the creation of objective data sources) when trying to meet everyone’s needs. There was no guarantee that our interviewees could or would always reach consensus when trying to agree on shared data structures, syntax, and semantics (Cheng et al., 2024). Fortunately, harmonization is just one of many strategies that can be used to help FIP researchers meet their needs. This is particularly important because we also found there is no agreement about what data needed to be harmonized in the first place. Our interviewees discussed when harmonization does not work: (1) when political, organizational, or institutional systems prevent harmonization from taking place, (2) when economic issues prevent data harmonization, (3) when the syntax or type of datasets is incompatible, (4) when the structure of data are difficult to identify and transform to allow interoperability, and (5) when the semantics of data are sufficiently different that they render comparisons meaningless or of limited value.

## CONCLUSIONS

This QI project evaluated the information needs of researchers from a diverse set of disciplines who rely on often sensitive, disparate, and difficult-to-obtain data. An influx of federal funding has provided researchers across the social sciences and health professions with an opportunity to collect new data and conduct needed research. However, data archivists and others supporting FIP researchers also must understand their needs. This paper provides insight into those needs.

The CVFP has created an opportunity to produce a harmonized dataset focused on public health interventions that prevent gun violence. The project, as it stands, only meets the needs of a narrow swath of users who study public health. We also spoke with criminologists who specialize in FIP. The two groups shared some experiences (e.g., frustration with the lack of reliable and timely information accompanied by data documentation), but the criminologists required different data. Criminologists used administrative data (secondary research), while health researchers created entirely new datasets to measure and test the effect of interventions. The usefulness of any system or systems supporting future cross-disciplinary data discovery and reuse will depend on many factors.

To meet FIP researcher information needs, a thriving data ecosystem must exist. Creating a thriving ecosystem will require collaboration among researchers, data managers, hospitals, government employees, and stakeholders across the data lifecycle. Researchers and data creators must be willing to contribute and provide documentation about their data. Coordination between disciplines that conduct FIP research will require adequate funding. Some data that researchers describe as resources (e.g., news) will likely never be provided in a database, dashboard, or even an archive. However, others (e.g., socioeconomic status and neighborhood crime rates) could bolster research capabilities by showing patterns between variables and across geographic regions.

Research is expensive and time-consuming, and grants have historically been the lifeblood of public health research. Perhaps the best that can be offered right now is an online resource (e.g., a guide coordinated by subject-matter experts and produced by a national agency such as the National Library of Medicine) that documents hard-to-find datasets and resources available to researchers. Many of the resources our interviewees discussed were not openly available or easy to use, and others required users to transform or modify datasets for their projects. From an information science perspective, more people are needed to process data and assist users in finding these sources. However, a more fundamental problem is that a significant amount of data is not being collected and coded in the first place. Thus, federal agencies and other funders should support the creation of a network coordinated by stakeholders across disciplines that includes a comprehensive guide to existing resources and has the technical infrastructure to support data standardization, documentation, and integration. This network may resemble or be a future instantiation of the CFVP.

While several participants discussed their need for a statewide or national set of resources, including dashboards that incorporate social, legal, and health factors, they also indicated that the effort required to create and maintain them is daunting. They said the data they deal with is messy, and it is not always possible to force data creators to adhere to any one set of formatting standards. However, this has been accomplished at the state level and may be possible elsewhere.

Unfortunately, research is often politicized, and public health research is particularly vulnerable to politicization. From disease prevention (e.g., vaccines and masking during COVID-19) to harm reduction (e.g., vehicle safety and Narcan training), skeptics and opponents often stymie public health efforts. FIP research has been inextricably associated with advocacy against firearms, but the epidemic of gun violence is real, and prevention is key to stopping it. The expertise of researchers, hopefully, can identify the root causes of America’s epidemic of firearm violence and provide resources and a way out of situations that cause people to turn to violence in the first place.

## Figures and Tables

**Table 1:** Themes and examples of quotes they describe.

THEME	INTERVIEW QUOTE
Some FIP Problems Are not Unique	‘[It’s…] not the context they’re from […] I think about what’s appealing or possible [… and] the incentives of the profession are set up to do […whatever is] quick. It’s easy [to use just whatever data…] you can get your hands on, but I think that affects more fields […]. I think it’s the doom loop we might have in terms of […incentives for the…] profession.’
Many Types and Sources of Data Exist	‘I’ve mostly been focusing on the secondary data analysis. Some of the data sets I’ve used […] are Add Health, the National Longitudinal Study of Adolescent to Adult Health… [the] National Survey of Child and Adolescent Wellbeing… But then, sometimes I just like… I need a statistic for a grant application or a statistic for a paper.’
Creatively Combining Data Can Cause Problems	‘A lot of the public health people who are coming in to look at gun violence don’t have the social science background… and they are missing some pretty key control variables or things that criminologists have known for decades… or they use data from criminology that they are not familiar with, and there are fatal flaws.’
Political Constraints Are Often Manageable	‘In Colorado [… they have a] contractor who’s a data science person. They have all the linked data… whether or not you have SNAP, whether you got shot, whether you had criminal justice involvement… Colorado’s well-funded, super motivated.’
Not All Researchers Want to Share Their Data	‘I submitted a FOIA request three years ago asking for something I know they have and that [is…] on their website. There are other public health researchers that have this data… [but…] they won’t share it with you unless they are a co-author.’
Data Harmonization is Contentious	‘[They are…] deciding which measures they will harmonize, for the six study teams… There are some unique constructs… I don’t think they can mandate, because for the harmonizing process, it’s like all the six projects, study teams, have to decide that they want to use it and then adopt […each measure].’
The Necessity of Grants	‘If we get the funding, we’re going to collect the clinical data, which we’ve harmonized… I don’t even know if that’s going to be hard because we haven’t started to do that, because we haven’t even applied for that part of the grant yet.’
Commonalities: The Needed Research Environment	‘I’ll say the three points, and ideally, in the future, the data. Number one is more current data. Second is more restricted data. Even the health department’s data is two years behind. The third one is the data linkage. We have all the data pieces, but […] we can’t link together to see a comprehensive picture.’

**Table 2:** Data resources by category.

CATEGORY	DATA SOURCE
**Health Data**	Behavioral Risk Factor Surveillance System (BRFSS)
	CDC Center for Injury Prevention
	CDC WISQARS (Web-based Injury Statistics Query and Reporting System)
	CDC WONDER
	Michigan emergency department data (bought the data)
	National Readmissions Database
	National Trauma Database
	TQIP (Trauma Quality Improvement Program)
	UMC Trauma Center Data
**Crime Data**	CJARS (Criminal Justice Administrative Records System)
	Domestic Violence Surveillance (DSVS)
	FBI Supplementary Homicide Reports
	Gun Violence Archive
	ICPSR Firearm Data Repository
	NACJD (National Archive of Criminal Justice Data)
	National Crime Victimization Survey (NCVS)
	NIBRS (National Incident-Based Reporting System)
	National Violent Deaths Reporting System
	Police department data
	Rapid Employment and Development Initiative (READI) Chicago
	School Crime Supplement (SOC)
	UCR (Uniform Crime Reporting Program)
**Social and Demographic Data**	American Community Survey
	Add Health (National Longitudinal Study of Adolescent to Adult Health)
	Childhood Opportunity Index
	FACTS National Survey
	FYI Study, Panel survey data
	National Firearm Attitudes and Behaviors Study
	NSCAW, National Survey of Child and Adolescent Wellbeing
	Social Vulnerability Index
	Youth Risk Behavior Surveillance System
**Educational Data**	Department of Education’s data
	Michigan Child Welfare System
**Custom Data**	Created own dataset
	Court documents
	Electronic Medical Records (from their hospital system to track admissions of gunshot injuries)
	Local shelters, next of kin interviews
	Police files, medical examiner data
	Toxicology reports
**News and Media Data**	AP News
	Google
	Newspapers
	USA Today

## Footnotes

1 Our search received support from the Director of the National Archive of Criminal Justice Data.

## APPENDIX A. SEMI-STRUCTURED INTERVIEW PROTOCOL

### BACKGROUND AND DEMOGRAPHICS

Instructions: Ask every participant these questions.

- Where do you work?
  - What is your current position or role?
- Which academic discipline (e.g., public health) are you most closely affiliated with?
  - Approximately how many years have you worked in this field?
- What research methods do you use?
- How do you get data for your research related to firearm violence? For example, you might collect your own or use administrative data.
- How do you use data other than for research?
- What else should I know about you (e.g., age or education)?

### COORDINATING CENTER

Instructions: Ask the Center for Firearm Violence Prevention (CFVP) based researchers.

- Which CFVP study site are you affiliated with?
- Can you describe the research aims and objectives, questions, and other issues addressed at this site?
- What methods are used at this site?
- What data is needed?
  - Are you directly collecting this data or not? Please elaborate.
- What kinds of data problems have you run into with this project? Potential examples include:
  - The type of data I need does not exist.
  - The data I need is scattered across many locations or is not in the format I need to do my work.
  - There is no good documentation available for the data I need.
  - Data is not accessible to me.
- Envision the perfect dataset for your project site. What would it contain? What could you do with it?
- What data-related support do you need from CFVP? Examples might include:
  - Access to restricted or sensitive data.
  - Preparing data for long-term archiving and/or preservation.
  - Analytical support.
  - Secure infrastructure to store sensitive data.
  - Subject-matter expertise to navigate data-related issues.
- What kind of experiences have you had building data sets or databases?
- What parts of this database do you think should be open for citizen science, or open for researchers? Moreover, how would it be most useful?
- What are the advantages and disadvantages of how this is being approached—with multiple sites, working from scratch to build this data set?

### OTHERS

Instructions: Ask CFVP Principal Investigators (PIs), Institute for Firearm Injury Prevention (IFIP) staff, and criminologists.

- How does your work relate to the CFVP or firearm research?
  - Please describe that work (e.g., I manage study data as a CFVP Co-PI).
- How did you come to work in or adjacent to firearm injury prevention?
- Describe the data needed for research in this space.
  - How did you come to that belief?
- If you conduct research in this space, do you collect your own data?
  - Why?
- What kinds of data problems have you run into when doing firearm injury prevention research? Examples include:
  - The type of data I need does not exist.
  - The data I need is scattered across many locations or is not in the format that I need to do my work.
  - There is no good documentation available for the data I need.
  - Data is not accessible to me.
- What kinds of data problems do you think affect other researchers?
- To what extent do you think the problems other researchers face result from the limits of our national firearm data infrastructure?
- Envision the perfect dataset for your research. What would it contain, and what would you do with it?
